# Prevalence and Clinical Significance of Aural Symptoms in Vestibular Migraine with and Without Ménière’s Disease

**DOI:** 10.3390/jcm15051687

**Published:** 2026-02-24

**Authors:** Seungmin Kwak, Seong Hoon Bae, Sung Huhn Kim, Sang Hyun Kwak, Bo Gyung Kim

**Affiliations:** 1 Department of Otorhinolaryngology, Yonsei University College of Medicine, Seoul 03722, Republic of Korea; andrewkwak@yuhs.ac (S.K.); bshsap1@yuhs.ac (S.H.B.); fledermaus@yuhs.ac (S.H.K.); 2 Department of Otorhinolaryngology, St. Vincent Hospital, College of Medicine, The Catholic University of Korea, Suwon 16247, Republic of Korea

**Keywords:** vestibular migraine, Ménière’s disease, aural symptoms, headache, dizziness, sensorineural hearing loss, tinnitus

## Abstract

**Objectives**: This study investigated the clinical characteristics of patients with vestibular migraine (VM) with a specific focus on aural symptoms. Additionally, we explored the distinct features of patients with VM who were also diagnosed with Ménière’s disease (MDVM). **Methods**: In this retrospective cohort study conducted in a tertiary center from 2016 to 2020, we analyzed clinical features, audiovestibular test results, and symptom improvement following medical therapy in 169 patients with VM. Logistic regression was used to identify factors linked to symptom improvement. Data collected from patients with VM and MDVM were compared. **Results**: Patients with VM commonly experienced aural symptoms, with 47.9% experiencing ear fullness, 40.2% reporting tinnitus, and 17.2% experiencing hearing fluctuations. Logistic regression analysis revealed that aural symptoms were predictive of a lower probability of symptom improvement for headache (*p* = 0.005) and dizziness (*p* = 0.007). Eleven (6.5%) patients with VM with aural symptoms were diagnosed with MD and exhibited distinct hearing patterns. The patients with MDVM showed a greater drop at low frequencies (mean hearing threshold of 33.6 ± 6.7 dB vs. 20.6 ± 11.1 dB in MDVM and VM, respectively; *p* = 0.002) and aggravated hearing levels on final audiometry (30.8 ± 23.0 dB vs. 12.6 ± 7.1 dB, *p* = 0.027). **Conclusions**: Aural symptoms are common among patients with VM and are associated with a lower probability of migraine symptoms responding to medication. Patients with MDVM exhibit a distinct pattern of hearing loss, leading to deterioration, whereas other patients with VM and aural symptoms show preserved hearing levels.

## 1. Introduction

Vestibular migraine (VM) is characterized by recurrent episodes of vestibular symptoms associated with migraine and other migrainous features, including headache, photophobia, phonophobia, and visual aura. Its diagnostic criteria are defined by the International Classification of Headache Disorders (ICHD-3) [1,2,3]. The annual prevalence of VM is estimated at 2.7%, making it one of the most common causes of dizziness [4]. The clinical manifestations of VM can be highly variable, and aural symptoms such as aural fullness, tinnitus, hearing loss, and otalgia are common [3,4,5]. These symptoms may overlap with those of Ménière’s disease (MD), which presents with recurrent vertigo, fluctuating hearing loss, aural fullness, and tinnitus [6,7,8,9]. Moreover, headache has been reported in a considerable proportion of patients with MD, further complicating the differential diagnosis between the two conditions. Emerging evidence also suggests that interactions within the trigeminovestibular system may contribute to the shared pathophysiological mechanisms of VM and MD [10,11].

The relationship between VM and MD has attracted considerable academic interest. Migraines are more common in patients with MD. Patients with MD exhibit a lifetime prevalence of migraine that is twice as high as that in the general population, reaching up to 56% [12,13]. The coexistence of the two diseases (MDVM) was found to comprise as much as 25% of the subjects who had either VM or MD [14]. Possible connection and commonalities in pathophysiology have been suggested, such as shared genetic susceptibility [15], underlying channelopathy [16], and possible inner ear damage related to migraine [13,17]. However, the definitive pathophysiological relationship between VM and MD remains unclear.

Although aural symptoms may occur in patients with VM, development of severe hearing loss is not expected [18,19,20]. In contrast, MD can result in a drastic decline in hearing and vestibular function [21]. The severity and audiometric patterns of sensorineural hearing loss differ between vestibular migraine and Ménière’s disease. In vestibular migraine, high-frequency sensorineural hearing loss of variable severity has been reported. In contrast, Ménière’s disease typically presents with low-frequency sensorineural hearing loss in the early stages, which may later progress to involve the entire frequency spectrum, usually in a unilateral pattern. These differences may provide useful clues in the differential diagnosis between the two conditions [9].

The treatment options are different between VM and MD. While lifestyle modifications are recommended for both, completely different types of medications are used. Occasionally, more aggressive measures are considered for MD, including intratympanic steroid injection and intratympanic aminoglycoside injection for the ablation of vestibular hair cells, and surgical options, such as endolymphatic sac surgery or vestibular neurectomy, for intractable cases. Thus, accurate differential diagnosis between VM and MD is essential for appropriate treatment. However, distinguishing patients with VM from those with early-stage MD who do not exhibit significant hearing loss during the nonictal phase remains challenging. The presence of aural symptoms in VM is the primary factor that complicates the diagnostic process because it mimics MD.

This study investigated the prevalence and clinical significance of aural symptoms in patients with VM differences in clinical symptoms and audiovestibular test results between VM and MDVM.

## 2. Materials and Methods

### 2.1. Patient Enrollment Criteria

**Figure 1:**
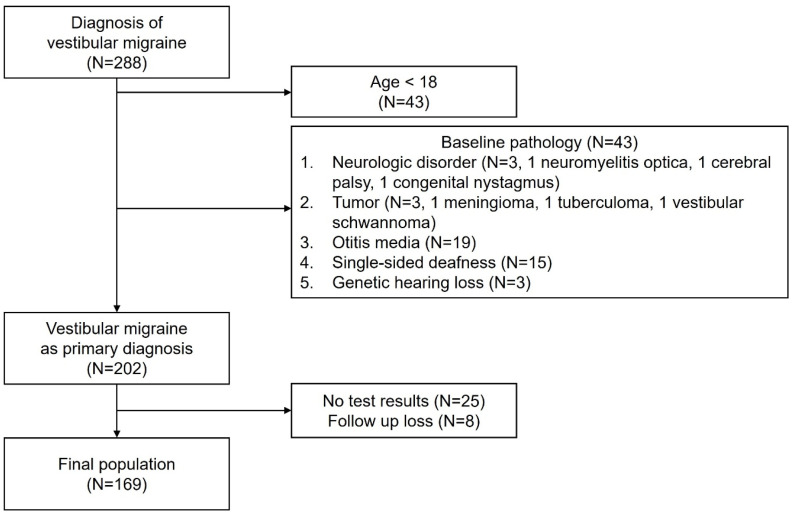
Diagram of patient selection.

A retrospective review of 288 patients diagnosed with VM at a single tertiary center (Department of Otorhinolaryngology, Yonsei University.) between 2016 and 2020 was conducted. The diagnosis of VM was established in accordance with the ICHD-3 [2]. These criteria include at least five episodes of vestibular symptoms of moderate or severe intensity lasting between 5 min and 72 h, a current or previous history of migraine with or without aura, and the presence of migrainous features (such as headache with migraine characteristics, photophobia, phonophobia, or visual aura) during at least 50% of vestibular episodes, with symptoms not better accounted for by another diagnosis. The exclusion criteria were (1) age < 18 years, (2) presence of central neurologic or other otologic pathology that could potentially alter the disease course, (3) follow-up loss before proper treatment, (4) unavailable audio-vestibular test results, and (5) history of prior treatment that could significantly affect baseline audiovestibular findings before the initial evaluation. The patient selection process is shown in Figure 1. This study was approved by the Institutional Review Board of Yonsei University (protocol code 4-2024-0059; approved on 12 March 2024).

### 2.2. Assessment of Clinical Symptoms

All patients underwent basic neurological and neurootological examinations conducted by experienced neurootologists. Brain magnetic resonance imaging was performed when clinically indicated to exclude intracranial lesions associated with central vertigo. The collection of data encompassed variables such as the age of onset and character of dizziness. Vertigo-type dizziness was defined as a perceptible spinning or rotational sensation, while other forms of dizziness, such as lightheadedness or unsteadiness, were categorized as non-vertigo. Also recorded were associated aural symptoms (aural fullness and tinnitus), presence of nystagmus, and characteristics of headache (as included in the diagnostic criteria for VM: unilaterality, pulsatile nature, presence of visual aura, severity of headache, photophobia, phonophobia, aggravation by physical activity, and nausea/vomiting).

### 2.3. Audio-Vestibular Testing

#### 2.3.1. Pure-Tone Audiometry (PTA)

Otoscopic examination and tympanometry were performed prior to audiometric testing to exclude external or middle ear pathology. Pure-tone audiometry (PTA) was conducted in a sound-attenuated booth using a clinical audiometer (AD629 audiometer, Interacoustics, Assens, Denmark) with standard supra-aural earphones. Air- and bone-conduction thresholds were measured at conventional frequencies according to standard clinical protocols, and masking was applied when appropriate. Hearing thresholds were expressed in decibels hearing level (dB HL).

The hearing threshold was calculated as the average of the thresholds at 0.5, 1, 2, and 4 kHz, and the degree of low-frequency hearing fluctuation (dB) was calculated as the average thresholds at 250 and 500 Hz. In cases of bilateral hearing loss and/or fluctuations, the threshold average for the worse ear was used. A change in hearing level of >5 dB was considered meaningful. Sensorineural hearing loss and its severity were classified according to established criteria [22].

At the time of enrollment, patients did not demonstrate definite sensorineural hearing loss characteristic of established Ménière’s disease. Baseline hearing levels were generally within the normal to mild hearing loss range according to the World Health Organization (WHO) classification of hearing impairment [22]. None of the patients showed definite audiometric progression consistent with Ménière’s disease at the initial evaluation. Patients who were subsequently diagnosed with Ménière’s disease were identified during the follow-up period based on the development of hearing fluctuations and fulfillment of the diagnostic criteria for definite Ménière’s disease. Therefore, the present study focused on patients evaluated prior to the development of clinically significant hearing deterioration.

#### 2.3.2. Bithermal Caloric Test

Otoscopic examination and tympanometry were performed prior to caloric testing to exclude external or middle ear pathology. The bithermal caloric test was performed using a caloric irrigator (Micromedical Technology Inc., Calabasas, CA, USA), and eye movements were recorded with an infrared video-based nystagmography system (Micromedical Technology Inc., Calabasas, CA, USA). Each ear was irrigated with water at 30 °C and 44 °C for 30 s. The maximum slow-phase velocity of nystagmus was calculated after each irrigation, and the Jongkees formula was used to determine canal paresis (CP). A CP value > 20% was considered abnormal.

#### 2.3.3. Video Head Impulse Test (vHIT)

The vHIT was performed for all six semicircular canals using a portable high-frame-rate video-oculography device (ICS Impulse, Otometrics, Denmark) comprising lightweight infrared goggles with built-in rate and acceleration sensors. The patients were instructed to visually fixate on a laser dot on a screen at a distance of 90 cm, and approximately 20 horizontal head impulses were manually applied to each side with unpredictable timing and direction. The peak head velocity of the impulses was maintained between 150 and 200°/s. The mean vestibulo-ocular reflex gains were automatically calculated. A horizontal VOR gain < 0.8 and, vertical VOR gain < 0.7 was considered abnormal based on previously reported normative data [23].

#### 2.3.4. Vestibular-Evoked Myogenic Potentials (VEMP)

Cervical and ocular vestibular-evoked myogenic potential (cVEMP and oVEMP, respectively) responses were recorded in the ipsilateral sternocleidomastoid muscle (cVEMP) or the contralateral inferior oblique ocular muscle (oVEMP) using 95 dB HL and 500 Hz tone burst stimulation (ABaer, Natus Medical, Inc., Pleasanton, CA, USA). Interaural ratio ≥ 0.4 for cVEMP, and ≥0.33 for oVEMP were considered abnormal [24,25,26].

#### 2.3.5. Electrocochleography (ECoG)

ECoG was conducted using the extratympanic method with 95 dB click stimuli, followed by calculation of the summating potential to action potential (SP/AP) ratio. An SP/AP ratio > 0.4 was considered abnormal [27,28]. The tests were performed using the protocols previously reported [29]. Vestibular function tests were performed at the initial visit to the outpatient clinic and follow-up tests were not performed, whereas PTA was regularly repeated over 3–6 months in patients with aural symptoms.

### 2.4. Measurement of Treatment Outcome

In patients with a migraine attack frequency of more than 1–2 times/week, a single or combination of suitable prophylactic medications such as anti-epileptics, beta-blockers, tricyclic antidepressants, and/or calcium channel blockers were used considering their response to initial medication and compliance, and additional as needed medication was used to control their headache and dizziness attacks. The others received medication as needed only to control their headaches and dizziness. Clinical outcomes were analyzed, and whether the symptoms improved following therapy was determined. Symptom improvement was defined as the relief of symptoms to the extent that allowed tapering of the prophylactic medication. If the frequency of dizziness and headaches decreased by >50% over 3 months, the disease was considered to have improved. The medication dose was then tapered.

### 2.5. Diagnosis of MDVM

For patients who experienced hearing fluctuations, diagnosis of MD was made based on the 2015 consensus document on diagnostic criteria from the Bárány Society [30]. Definite MD was defined as: (1) two or more spontaneous episodes of vertigo each lasting 20 min to 12 h; (2) audiometrically documented low- to mid-frequency sensorineural hearing loss in the affected ear on at least one occasion before, during, or after one of the episodes of vertigo; (3) fluctuating aural symptoms (hearing loss, tinnitus, or fullness) in the affected ear; and (4) symptoms not better accounted for by another vestibular diagnosis. The patients were regarded as having MD when the “definite MD” criteria were met at any time point during the follow-up period. The latest PTA results of these patients were collected for comparison.

### 2.6. Statistical Analysis

Statistical analysis was conducted using SPSS software (version 18.0; SPSS Inc., Chicago, IL, USA). Continuous variables were tested for normality using the Shapiro–Wilk test. Multivariate logistic regression analysis was performed to identify the factors associated with symptom improvement after medical therapy. Variables selected for logistic regression included sex, age, and other factors (*p*-value < 0.1 in the univariable analysis). These variables included dizziness type (vertigo vs. non-vertigo), associated ear symptoms (tinnitus or ear fullness), and visual aura. To control for confounding factors, tinnitus and ear fullness were considered as a single variable. The variables for dizziness improvement included dizziness type, associated tinnitus, and aural fullness. Model validity was assessed using the Hosmer–Lemeshow goodness-of-fit test. Furthermore, the data of patients with MDVM were compared with those of patients with VM who did not have MD but presented with aural symptoms. The chi-squared test, Fisher’s exact test, two-sample *t*-test, and Mann–Whitney U test were used as appropriate. A two-tailed *p*-value < 0.05 was considered statistically significant.

## 3. Results

### 3.1. Demographics and Symptoms Related to VM

In total, 169 patients were enrolled (Figure 1). There was a significant female preponderance, with 20 (11.8%) males and 149 (88.2%) females (*p* < 0.001). The average follow-up period was 59.4 months (range: 40.2–164.2). Among the enrolled patients, 121 (71.6%) experienced vertigo-type dizziness, and 30 (17.8%) displayed either spontaneous or evoked nystagmus. Visual aura was present in 38 (22.5%), photophobia in 47 (27.8%), and phonophobia in 63 (37.3%) patients. These data are summarized in Table 1.

### 3.2. Vestibular Function Test Results

Vestibular function test results were mostly within the normal range (Table 1). In the caloric test, 12 (7.1%) patients showed CP greater than 25%. Two patients were diagnosed with definite MD. Among the 80 patients who underwent ECoG testing, four (5.0%) had an elevated SP/AP ratio (>0.4). Only one patient (11.1% of patients with MDVM who underwent ECoG) was diagnosed with definite MD. Of the 153 individuals who underwent the vHIT, only four (2.6%) exhibited abnormalities. One patient showed decreased gain in the posterior semicircular canal, and three displayed catch-up saccades with normal gain, involving the lateral semicircular canal in two patients and the posterior semicircular canal in one patient.

### 3.3. Audiometry and Prevalence of Aural Symptoms

All patients had a normal average baseline hearing level. The mean hearing threshold on PTA was 12.1 ± 8.8 dB. Despite having normal hearing, a notable number of patients reported accompanying aural symptoms; specifically, 81 (47.9%) experienced aural fullness and 68 (40.2%) reported tinnitus. Hearing fluctuation, which manifested on PTA as a transient increase in the low-frequency threshold, was observed in 29 (17.2%) cases. In these patients, there was an average increase of 25.5 ± 11.6 dB at low frequencies (250 Hz and 500 Hz). Overall, 92 patients (54.4%) had aural symptoms, and 29 patients (31.5%) exhibited hearing fluctuations (Table 1).

### 3.4. Aural Symptoms as Prognostic Factors for Headache and Dizziness

Following appropriate medical therapy, the overall number of headache and dizziness was significantly reduced from 1.60 ± 0.71/week to 0.90 ± 0.81/week, and 1.53 ± 0.69/week to 0.80 ± 0.77/week, respectively (*p* < 0.001). Headache showed a statistically significant improvement in 112 cases (66.3%; from 1.48 ± 0.71/week to 0.39 ± 0.29/week, *p* < 0.001), and dizziness also improved significantly in 116 cases (68.6%; from 1.53 ± 0.73/week to 0.39 ± 0.35/week, *p* < 0.001) (Table 1). After univariate analysis (Supplementary Tables S1 and S2), logistic regression analysis was performed to identify the factors associated with the likelihood of improvement in headache and dizziness (Table 2 and Table 3). Non-vertigo type of dizziness (odds ratio (OR) 95% confidence interval (CI) 0.13 to 0.69; *p* = 0.005) and aural fullness or tinnitus (OR 95% CI 0.15 to 0.71; *p* = 0.005) were identified as statistically significant predictors of lower probability of improvement regarding headache. Similarly, for dizziness, non-vertigo type of dizziness (OR 0.31, 95% CI 0.14–0.71; *p* = 0.005) and the presence of associated aural symptoms (aural fullness or tinnitus) (OR 0.34, 95% CI 0.16–0.75; *p* = 0.007) were identified as statistically significant predictors of a lower probability of improvement. Therefore, the presence of aural symptoms can serve as an indicator of persistent headache and dizziness after medical therapy in VM.

### 3.5. Comparison Between Patients with MDVM or VM

Among the 92 patients who complained of aural symptoms, 29 (31.5%) showed fluctuations in hearing on PTA, and 11 (12.0%) were diagnosed with definite MD (MDVM) (Table 1). Sex, age, aural symptoms, and other migraine features did not significantly differ between the VM and MDVM groups. Furthermore, there was no significant difference in the vestibular test results (Table 4). Patients with MDVM exhibited a distinct pattern of hearing fluctuations compared to patients with VM with hearing fluctuations. Although both groups had hearing fluctuations in the low frequencies (250 Hz and 500 Hz), patients with MDVM demonstrated a greater drop in hearing from the baseline (mean hearing threshold at 250 and 500 Hz of 33.6 ± 6.7 dB vs. 20.6 ± 11.1 dB in MDVM and VM, respectively; *p* = 0.002). Patients with VM with hearing fluctuations maintained normal hearing thresholds on the final PTA (mean 12.6 ± 7.1 dB, median follow-up period of 50.7 months, interquartile range (IQR) 46.8 to 94.7), with no significant difference compared to the initial baseline PTA threshold (mean 12.5 ± 8.1 dB, *p* = 0.945). In contrast, patients with MDVM had significant hearing loss by the last follow-up date (mean 30.8 ± 23.0 dB, *p* = 0.027, median follow-up period of 70.1 months, IQR 39.0 to 109.7) (Table 3).

## 4. Discussion

We investigated the clinical characteristics of patients with VM, focusing on the aural symptoms and hearing fluctuations. The distinction between VM and MD is an area of ongoing research. The factors suggested in previous studies that favor MD over VM include older age, male sex, prolonged duration of dizziness, presence of sensorineural hearing loss, absence of migraine history, and lack of features such as aura, photophobia, and phonophobia [8,14]. In particular, fluctuating low-frequency sensorineural hearing loss, which is characteristic of Ménière’s disease, represents one of the most important clinical criteria for differential diagnosis between the two disorders [9]. Abnormal findings on audio-vestibular tests, including audiometry, VEMP, and ECoG, have been reported to be more associated with MD than with VM [8,9,14,26,27,31,32,33,34,35]. In addition, vestibular function tests such as caloric testing and the video head impulse test (vHIT) have differential diagnostic value, as objective signs of peripheral vestibular dysfunction are more frequently observed in Ménière’s disease than in vestibular migraine [36]. Such observations align with the progressive deterioration of inner ear function seen in patients with MD, where the differences become more pronounced as the disease advances. However, the significance of these differences in the early stages of the disease remains unclear. Furthermore, few studies have explored the distinct clinical characteristics associated with the coexistence of VM and MD. To the best of our knowledge, our study is the first to evaluate patients with MD during follow-up, when audiometric findings were still within the normal to mild hearing loss range at baseline, with the aim of identifying factors that could aid in the differential diagnosis of VM and MD.

The clinical distinction between vestibular migraine and Ménière’s disease has long been recognized as challenging because of substantial overlap in episodic vertigo, fluctuating auditory symptoms, and associated migraine features. Previous studies have reported that a considerable proportion of patients initially diagnosed with one disorder may later fulfill diagnostic criteria for the other during follow-up, suggesting either phenotypic overlap or potential shared pathophysiological mechanisms [3,13,14,30]. In particular, differentiation is most difficult in the early stages of disease, when audiometric abnormalities may be subtle or absent and vestibular testing results are often inconclusive [13,30]. These diagnostic uncertainties underscore the importance of identifying early clinical indicators that may help distinguish between the two conditions.

The diagnostic process is sometimes challenging because of the overlapping clinical symptoms of VM and MD. In our study, 71.6% of all patients with VM experienced true vertigo and 17.8% had clinically observed nystagmus. Over half (54.4%) of the patients with VM in our study reported aural fullness or tinnitus associated with headaches or dizziness. Nearly one-third of patients (31.5%) displayed hearing fluctuations, mainly at frequencies below 1 kHz. Moreover, we found that the presence of aural fullness or tinnitus indicated prolonged symptom severity and the need for medication. Patients with aural symptoms had a lower chance of headache improvement after medical therapy. Logistic regression analysis revealed that tinnitus and aural fullness predicted a significantly lower probability of headache improvement. Aural fullness was also associated with a lower chance of dizziness improvement. Therefore, the differential diagnosis in such patients can be even more confusing owing to persistent VM symptoms, which may lead to inappropriate treatment.

Additionally, the absence of vertigo-type dizziness was identified as a predictor of a lower chance of improvement in both headaches and dizziness. The reason patients with true vertigo respond better to medications remains uncertain. One possible explanation is the potential for more direct involvement of the central vestibular pathway targeted by medication. As there are no established guidelines supported by evidence specifically for the treatment of VM [37,38], further research is warranted to elucidate the factors associated with clinical improvement.

Patients with VM exhibited normal initial audiometry and vestibular function, similar to patients with MDVM. A decline in audiovestibular function is not common in VM [18,19,20]. In this study, patients with VM without MD maintained normal hearing levels, and even those with hearing fluctuations did not experience further deterioration. Conversely, patients with MDVM experience a gradual decrease in hearing levels, leading to significantly worse final PTA results. While certain predisposing factors or pathogenic pathways may be shared between VM and MD [7,13], the diagnosis of MD reflects an immediate pathological process in the inner ear. None of the other patients with VM with aural symptoms experienced a significant decline in hearing, which aligns with the well-established favorable prognosis of VM. Notably, the degree of hearing fluctuation at low frequencies was greater in patients with MDVM than in those with VM. This appears to be a distinct characteristic of MDVM in contrast to VM.

In MD, sensorineural hearing loss may precede the onset of vertigo by several months or years in the form of delayed hydrops. Conversely, tinnitus or aural fullness is typically associated with the first episode of vertigo [3]. In the present study, patients who were subsequently diagnosed with MDVM consistently reported aural symptoms from the initial clinical evaluation, suggesting that early auditory complaints may represent an important clinical indicator of underlying inner ear pathology. In contrast, patients with VM without aural symptoms did not demonstrate progression to hearing loss during follow-up. In a study regarding the long-term prognosis of VM, the proportion of patients with concomitant aural symptoms increased from 16% to 49% over 5.5–11 years of follow-up, and none of them developed a severe form of hearing loss indicative of inner ear pathology [18]. Although patients with VM without aural symptoms may eventually experience tinnitus or aural fullness, the progression to clinically significant sensorineural hearing loss appears to be rare, with no cases reported during long-term follow-up in previous cohorts [18].

In previous investigations [14] migrainous traits, such as aura, photophobia, and phonophobia, were reported to occur less frequently in the MD population than in the VM population. In our study, patients with MDVM exhibited a lower incidence of visual aura, photophobia, and phonophobia. Despite these observations, statistical significance was not achieved, and these traits do not seem to offer a satisfactory reference for clinical decision making.

Abnormal findings on caloric tests, VEMP, and EcoG are more commonly associated with MD than VM [8,9,14,26,27,31,32,33,34,35]. However, in our study, vestibular testing failed to distinguish MDVM from VM. Although slightly higher caloric CP and ECoG SP/AP ratios were noted in patients with MD, these differences were not statistically significant. This finding may be explained by the disease stage of our cohort, as vestibular hypofunction is typically observed in more advanced stages of Ménière’s disease, whereas objective vestibular function may remain normal during the early phase. Therefore, the absence of significant abnormalities in vestibular testing in our study population is not unexpected. Despite its established role in the evaluation of MD, vestibular testing alone may have limited diagnostic value for detecting early-stage disease.

Our study had several limitations. This was a retrospective study conducted at a single center, which may have affected the generalizability of our findings. When assessing symptom improvement in the patients, no effective parameters were available to quantitatively measure the degree of headache or dizziness. The lack of a standardized protocol for prescribing medication is notable, as decisions are made subjectively by individual clinicians depending on patient compliance, medication side effects, and individual symptom changes. The follow-up periods varied, and some patients did not have sufficient follow-up periods to support the conclusions. Recently, monoclonal antibodies targeting calcitonin gene-related peptide (CGRP) have emerged as effective preventive therapies for migraine [39]. Because the preventive medications used in this study were heterogeneous and did not include anti-CGRP agents, the observed poorer treatment response in patients with aura should be interpreted with caution. Prospective studies with standardized treatment protocols, including anti-CGRP therapy and uniform follow-up periods, are warranted to validate our findings.

From a practical clinical perspective, differentiating VM from MD remains challenging, particularly in the early stages when audiovestibular findings may be subtle. Current diagnostic guidelines suggest that progressive or fluctuating sensorineural hearing loss and objective vestibular hypofunction favor MD, whereas migrainous features with stable hearing are more suggestive of VM. However, overlap between the two conditions is common, and longitudinal observation may be required for definitive diagnosis. A practical summary of distinguishing features based on current evidence and the findings of this study is provided in Table 5.

## 5. Conclusions

Aural symptoms, including tinnitus, aural fullness, and hearing fluctuations, are frequently observed in patients with VM and may complicate differentiation from MD. In contrast to VM, MDVM shows a larger degree of low-tone fluctuation and progression of eventual hearing deterioration, which may help distinguish MDVM from VM. These findings suggest that longitudinal audiometric monitoring, particularly focusing on low-frequency changes, may provide practical clinical value in distinguishing early MD from VM.

## Figures and Tables

**Table 1:** Demographics, clinical characteristics, audio-vestibular test results, and clinical outcomes of patients diagnosed with vestibular migraine.

	Total (N = 169)
*Demographics and clinical characteristics*	
Sex (male:female)	20:149
Age of onset, years	36.7 ± 13.7
Vertigo-type dizziness, *n* (%)	121 (71.6%)
Associated otologic symptoms, *n* (%)	92 (54.4%)
Aural fullness, *n* (%) Tinnitus, *n* (%)	81 (47.9%)
	68 (40.2%)
Presence of nystagmus, *n* (%)	30 (17.8%)
Migraine-related symptoms	
Visual aura, *n* (%)	38 (22.5%)
VAS score, 0 (none)–10 (severe)	6.9 ± 1.5
Photophobia, *n* (%)	47 (27.8%)
Phonophobia, *n* (%)	64 (37.9%)
Aggravation by physical activity, *n* (%)	71 (42.0%)
Nausea or vomiting, *n* (%)	134 (79.3%)
*Audio-vestibular test results*	
Baseline PTA threshold, dB HL	12.1 ± 8.8
Low frequency fluctuation on PTA, *n* (%), dBHL	29 (17.2%), 25.5 ± 11.6
Canal paresis on caloric test, % (N = 162)	11.4 ± 11.5
cVEMP abnormality (N = 158)	
Normal, *n* (%)	96 (60.8%)
Unilateral loss, *n* (%)	46 (29.1%)
Bilateral loss, *n* (%)	16 (10.1%)
oVEMP abnormality (N = 94)	
Normal, *n* (%)	23 (24.5%)
Unilateral loss, *n* (%)	26 (27.7%)
Bilateral loss, *n* (%)	45 (47.9%)
ECoG SP/AP ratio (N = 80)	0.27 ± 0.09
Clinical outcomes	
Headache improvement, *n* (%)	112 (66.3%)
Dizziness improvement, *n* (%)	116 (68.6%)
Diagnosis of definite MD, *n* (%)	11 (6.5%)

Continuous variables are presented as mean ± standard deviation (SD). The audiometry thresholds represent the four-frequency average at 500, 1000, 2000, and 4000 Hz. Low-frequency fluctuation is determined by calculating the average increase in threshold at 250 and 500 Hz, compared to the baseline. cVEMP: cervical vestibular-evoked myogenic potential, ECoG: electrocochleography, MD: Ménière’s disease, oVEMP: ocular vestibular-evoked myogenic potential, PTA: pure tone audiometry, SP/AP: summating potential to action potential ratio, VAS: visual analog scale.

**Table 2:** Logistic regression analysis of predictive factors for headache improvement after medical therapy.

	OR (95% CI)	*p*-Value
Male sex	0.41 (0.14–1.21)	0.107
Age	1.00 (0.97–1.02)	0.787
Non-vertigo	0.30 (0.13–0.69)	0.005 **
Associated aural fullness or tinnitus	0.33 (0.15–0.71)	0.005 **
Visual aura	0.67 (0.30–1.52)	0.337

A significance level of *p* < 0.05 was considered statistically significant. Variables with OR < 1 indicate a lower likelihood of headache improvement after medical therapy. CI, confidence interval; OR, odds ratio. **: *p*-value < 0.01.

**Table 3:** Logistic regression analysis of predictive factors for dizziness improvement after medical therapy.

	OR (95% CI)	*p*-Value
Male sex	0.65 (0.22–1.87)	0.420
Age	0.99 (0.97–1.02)	0.661
Non-vertigo	0.31 (0.14–0.71)	0.005 *
Associated aural fullness or tinnitus	0.35 (0.16–0.75)	0.007 *

The table displays variables associated with a lower likelihood of improvement. CI: confidence interval, OR: odds ratio. *: *p*-value < 0.05.

**Table 4:** Comparison between patients with or without a concurrent diagnosis of Ménière’s disease.

Total (N = 92)	Aural Fullness or Tinnitus, and Stable Hearing (N = 63)	Hearing Fluctuations, Non-MD (N = 18)	Definite MD (N = 11)	*p*-Value (MD vs. the Rest)
Sex (male:female)	6:57	3:15	2:9	0.616
Onset age, years	34.1 ± 13.2	33.9 ± 11.7	35.3 ± 12.9	0.763
Vertigo-type dizziness, *n* (%)	51 (81.0%)	12 (66.7%)	11 (100%)	0.114
Visual aura, *n* (%)	18 (28.6%)	5 (27.8%)	1 (9.1%)	0.277
Photophobia, *n* (%)	21 (33.3%)	3 (16.7%)	2 (18.2%)	0.722
Phonophobia, *n* (%)	28 (44.4%)	5 (27.8%)	2 (18.2%)	0.196
Baseline PTA threshold, dB HL	10.9 ± 8.5	12.5 ± 8.1	12.4 ± 7.9	0.682
Low frequency fluctuation, dB HL (during aggravation)		20.6 ± 11.1 **	33.6 ± 6.7 **	0.002 **
Final PTA threshold, dB HL		12.6 ± 7.1 *	30.8 ± 23.0 *	0.027 *
Canal paresis, % (N = 88)	11.1 ± 10.9 (N = 60)	15.0 ± 13.8 (N = 18)	19.4 ± 19.2 (N = 10)	0.097
cVEMP results (N = 88)	(N = 60)	(N = 17)	(N = 11)	0.252
Normal, *n* (%) Unilateral loss, *n* (%) Bilateral loss, *n* (%)	40 (66.7%)	11 (64.7%)	7 (63.6%)	
	17 (28.3%)	5 (29.4%)	2 (18.2%)	
	3 (5.0%)	1 (5.9%)	2 (18.2%)	
ECoG SP/AP ratio (N = 59)	0.27 ± 0.10 (N = 40)	0.29 ± 0.12 (N = 10)	0.30 ± 0.10 (N = 9)	0.229

The audiometry thresholds represent the four-frequency average at 500, 1000, 2000, and 4000 Hz. Low-frequency fluctuation is determined by calculating the average increase in threshold at 250 and 500 Hz, compared to the baseline. (*: *p*-value < 0.05, **: *p*-value < 0.01); cVEMP: cervical vestibular-evoked myogenic potential, ECoG: electrocochleography, MD: Ménière’s disease, PTA: pure tone audiometry, SP/AP: summating potential to action potential ratio.

**Table 5:** Practical considerations for differentiating vestibular migraine and Ménière’s disease based on current diagnostic guidelines.

Feature	Vestibular Migraine (VM)	Ménière’s Disease (MD)
Vertigo duration	5 min–72 h, variable	20 min–12 h, typically recurrent
Migraine history/features	Common (aura, photophobia, phonophobia)	Less common
Hearing loss	Usually absent, but minimal low-frequency hearing loss may occur and fluctuate	Fluctuating, progressive low-frequency SNHL
Aural symptoms	Tinnitus/fullness possible but often transient	Tinnitus/fullness with vertigo episodes, progressive
Audiometry over time	Stable	Deterioration over follow-up
Vestibular testing	Often normal	Peripheral hypofunction in advanced stages
Disease course	Favorable prognosis, limited cochlear damage	Progressive inner ear dysfunction

SNHL: sensorineural hearing loss. This summary is based on the diagnostic criteria proposed by the Bárány Society for Ménière’s disease and the International Headache Society classification for vestibular migraine (ICHD-3).

## Data Availability

The data presented in this study are available on request from the corresponding author due to patient privacy concerns and ethical restrictions.

## Supplementary Materials

The following supporting information can be downloaded at: https://www.mdpi.com/article/10.3390/jcm15051687/s1, Table S1: Univariate analysis of clinical factors associated with headache improvement; Table S2: Univariate analysis of clinical factors associated with dizziness improvement.

## Abbreviations

The following abbreviations are used in this manuscript:

VM	Vestibular migraine
MD	Ménière’s disease
ICHD-3	International Classification of Headache Disorders-3
PTA	Pure tone audiometry
CP	Canal paresis
vHIT	Video head impulse test
cVEMP	Cervical vestibular-evoked myogenic potential
oVEMP	Ocular vestibular-evoked myogenic potential
ECoG	Electrococleography
SP	Summating potential
AP	Action potential
OR	Odds ratio
CI	Confidence interval
