# Supplementary material for: Prevalence and Clinical Significance of Aural Symptoms in Vestibular Migraine with and Without Ménière’s Disease
Source: J Clin Med. 2026 Feb 24;15(5):1687. doi: 10.3390/jcm15051687 (PMC12986516; doi:10.3390/jcm15051687)
Supplement: Supplementary file 1 [file jcm-15-01687-s001.zip › jcm-4145500-supplementary.pdf]

Supplementary Table S1. Univariate analysis of clinical factors associated with headache improvement.

	**Headache improved (n=111)**	**Headache unimproved (n=58)**	**p-value**
Demographics and clinical characteristics			
Sex (male:female)	11:100	9:49	0.284
Age of onset, years	39.7±14.1	38.2±13.9	0.522
Vertigo-type dizziness, n (%)	84 (75.7%)	38 (65.5%)	0.162
Associated otologic symptoms, n (%)	**48 (43.2%)**	**39 (67.2%)**	**0.003**
Aural fullness, n (%)	**42 (37.8%)**	**35 (60.3%)**	**0.005**
Tinnitus, n (%)	**33 (29.7%)**	**30 (51.7%)**	**0.005**
Presence of nystagmus, n (%)	21 (18.9%)	8 (13.8%)	0.401
Migraine-related symptoms			
**Visual aura, n (%)**	**24 (21.6%)**	**21 (36.2%)**	**0.042**
VAS score, 0 (none) – 10 (severe)	6.8±1.5	7.2±1.5	0.168
Photophobia, n (%)	32 (28.8%)	14 (24.1%)	0.515
Phonophobia, n (%)	44 (39.6%)	19 (32.8%)	0.380
Aggravation by physical activity, n (%)	44 (39.6%)	26 (44.8%)	0.516
Nausea, n (%)	81 (73.0%)	53 (91.4%)	0.005
Audio-vestibular test			

results			
Baseline PTA threshold, dB HL	11.6±7.9	13.9±11.7	0.182
Low frequency fluctuation on PTA, n (%), dB HL	**12 (10.8%), 1.7±6.2**	**14 (24.1%), 4.4±9.6**	**0.023, 0.051**
Canal paresis on caloric test, % (N=162)	11.4±11.9	11.9±11.2	0.824
cVEMP abnormality (N=158)	(n=104)	(n=55)	0.102
Normal, n (%)	66 (63.5%)	31 (56.4%)	
Unilateral loss, n (%)	25 (24.0%)	21 (38.2%)	
Bilateral loss, n (%)	13 (12.5%)	3 (5.5%)	
oVEMP abnormality (N=94)	(n=64)	(n=30)	0.981
Normal, n (%)	16 (25.0%)	8 (26.7%)	
Unilateral loss, n (%)	18 (28.1%)	8 (26.7%)	
Bilateral loss, n (%)	30 (46.9%)	14 (46.7%)	
ECoG SP/AP ratio (N=80)	0.26±0.07	0.29±0.13	0.191

The chi-squared test, Fisher's exact test, two-sample t-test, and Mann–Whitney U test were used as appropriate. Factors with a p-value <0.1 were included in the logistic regression analysis. cVEMP: cervical vestibular-evoked myogenic potential; ECoG: electrocochleography, oVEMP: ocular vestibular-evoked myogenic potential; MD: Meniere's disease, PTA: pure tone audiometry; SP/AP: summating potential to action potential ratio, VAS: visual analog scale

Supplementary Table S2. Univariate analysis of clinical factors associated with dizziness improvement.

	**Dizziness improved (n=114)**	**Dizziness unimproved (n=55)**	**p-value**
Demographics and clinical characteristics			
Sex (male:female)	13:101	7:48	0.803
Age of onset, years	39.5±14.3	38.7±13.6	0.726
Vertigo-type dizziness, n (%)	87 (76.3%)	35 (63.6%)	0.085
Associated otologic symptoms, n (%)	**52 (45.6%)**	**35 (63.6%)**	**0.028**
Aural fullness, n (%)	**44 (38.6%)**	**33 (60.0%)**	**0.009**
Tinnitus, n (%)	**36 (31.6%)**	**27 (49.1%)**	**0.027**
Presence of nystagmus, n (%)	20 (17.5%)	9 (16.4%)	0.849
Migraine-related symptoms			
Visual aura, n (%)	26 (22.8%)	19 (34.5%)	0.106
VAS score, 0 (none) – 10 (severe)	6.8±1.6	7.2±1.3	0.205
Photophobia, n (%)	33 (28.9%)	13 (23.6%)	0.467
Phonophobia, n (%)	43 (37.7%)	20 (36.4%)	0.864
Aggravation by physical activity, n (%)	44 (38.6%)	26 (47.3%)	0.283
Nausea, n (%)	86 (75.4%)	48 (87.3%)	0.075
Audio-vestibular test			

results			
Baseline PTA threshold, dB HL	12.3±8.9	12.3±10.4	0.997
Low frequency fluctuation on PTA, n (%), dB HL	**13 (11.4%), 1.7±6.2**	**13 (23.6%), 4.4±9.6**	**0.039, 0.112**
Canal paresis on caloric test, % (N=162)	11.2±11.2	12.4±12.4	0.526
cVEMP abnormality (N=158)	(n=107)	(n=52)	0.820
Normal, n (%)	64 (59.8%)	33 (63.5%)	
Unilateral loss, n (%)	31 (29.0%)	15 (28.8%)	
Bilateral loss, n (%)	12 (11.2%)	4 (7.7%)	
oVEMP abnormality (N=94)	(n=63)	(n=31)	0.433
Normal, n (%)	32 (50.8%)	12 (38.7%)	
Unilateral loss, n (%)	15 (23.8%)	11 (35.5%)	
Bilateral loss, n (%)	16 (25.4%)	8 (25.8%)	
ECoG SP/AP ratio (N=80)	0.27±0.10	0.28±0.08	0.741

The chi-squared test, Fisher's exact test, two-sample t-test, and Mann–Whitney U test were used as appropriate. Factors with a p-value <0.1 were included in the logistic regression analysis. cVEMP: cervical vestibular-evoked myogenic potential; ECoG: electrocochleography, MD: Meniere's disease; oVEMP: ocular vestibular-evoked myogenic potential, PTA: pure tone audiometry; SP/AP: summating potential to action potential ratio, VAS: visual analog scale.
